# The Role of Cathepsin B in Pathophysiologies of Non-tumor and Tumor tissues: A Systematic Review

**DOI:** 10.7150/jca.86531

**Published:** 2023-07-24

**Authors:** Jiangping Wang, Minying Zheng, Xiaohui Yang, Xinyue Zhou, Shiwu Zhang

**Affiliations:** 1 Graduate School, Tianjin University of Traditional Chinese Medicine, Tianjin, 301617, P.R. China.; 2 Department of Pathology, Tianjin Union Medical Center, Tianjin, 300071, P.R. China.; 3 Nankai University School of Medicine, Nankai University, Tianjin, 300071, P.R. China.; 4 Graduate School, Tianjin Medical University, Tianjin, 300070, P.R. China.

**Keywords:** Cathepsin B, extracellular matrix, cell death, tumor microenvironment, epithelial-mesenchymal transition

## Abstract

Cathepsin B (CTSB), a lysosomal cysteine protease, plays an important role in human physiology and pathology. CTSB is associated with various human diseases, and its expression level and activity are closely related to disease progression and severity. Physiologically, CTSB is integrated into almost all lysosome-related processes, including protein turnover, degradation, and lysosome-mediated cell death. CTSB can lead to the development of various pathological processes through degradation and remodeling of the extracellular matrix. During tumor development and progression, CTSB has two opposing effects. Its pro-apoptotic properties reduce malignancy, while its proteolytic enzymatic activity promotes invasion and metastasis, thereby inducing malignancy. Here, we discuss the roles of CTSB in tumor and non-tumor disease pathophysiologies. We conclude that targeting the activity or expression of CTSB may be important for treating tumor and non-tumor diseases.

## Introduction

Proteases are a large group of enzymes that catalyze the cleavage of peptide linkages in proteins. Based on the substrate catalyzed by the enzyme and pH, proteolytic enzymes can be classified into four types: serine proteases, matrix metalloproteases, cysteine proteases, and aspartic proteases. Cathepsin B (CTSB), a lysosomal cysteine protease first reported in 1957, plays a role in intracellular proteolysis 1. In some cases, it may also be involved in other physiological processes, such as the processing of antigens to induce an immune response, hormone activation, and bone turnover 2. This protein is widely distributed in human tissues and is involved in many pathological processes 3. In non-tumor diseases, CTSB may act as a secretase involved in the secretory pathway regulated by brain neurons. CTSB plays a central role in extracellular matrix remodeling and is associated with the development of cardiovascular diseases. In addition, CTSB is highly expressed in the extracellular space and at the edge of the invading tumor to enhance the invasion and metastasis of cancer cells 4. CTSB also plays an important role in cell death 5. It is produced as a precursor (pro-CTSB) to CTSB and undergoes proteolytic processing and glycosylation to a mature form containing heavy and light chains. Intracellular CTSBs exist primarily in the mature form, and extracellular CTSBs primarily exist in the precursor form 2.

## The structure of CTSB

Structurally, CTSB is a bilobed protein with a distinct cut at the "top," representing the active site cleft. Its polypeptide chain folds into two distinct domains (the left "L" domain and the right "R" domain) that interact through an extended polar interface and open into a V-shaped active site cleft 6. The human CTSB gene is located on the short arm of chromosome 8 (8p22-p23.1) 7 and comprises 13 exons. The upstream region of the first exon is a 2.2 kb promoter region rich in GC base pairs but lacks the canonical TATA and CAAT boxes. There are six specificity protein 1 binding sites, four Ets protein binding sites, and one enhancer box (E-box) that regulate transcription, while specificity protein 1 and Ets1 proteins have been confirmed to enhance the transcription of CTSB 8. CTSB, as a proenzyme containing a signal sequence, is synthesized on the rough endoplasmic reticulum, where the CTSB propeptide is cleaved into an inactive 46 kDa form of pro-CTSB, which is then glycosylated before being transported to the Golgi, where it is transported in the form of an active 31 kDa single-chain via vesicular trafficking to late endosomes. Upon fusion of late endosomes with lysosomes to become endolysosomes, the mature single-stranded form is further processed into the active double-chain forms of 25/26 and 5 kDa^9^. CTSB exists in two forms: an inactive form (zymogen form) and an active mature form. There are two active forms of CTSB: the mature single-chain (31 kDa) isoform and the heavy chain of the mature double-chain isoform (25/26 kDa). Only mature CTSBs are biologically active.

## The function of CTSB

CTSB is a member of the cysteine protease family and there are three isoforms of CTSB including single-chain form (molecular mass is about 31 kDa), a heavy-chain form with a complete C-terminal end (25 kDa) and a heavy-chain form having a truncated C-terminal end (23.4 kDa) 10. Different CTSB isoforms have different subcellular localizations, which may determine their different functions and independent regulatory mechanisms 11. The main physiological function of CTSB is to participate in the lysosomal degradation of different proteins to maintain a stable state of the intracellular proteome 12. CTSB plays a central role in a variety of pathological processes, including initiation, proliferation, growth, angiogenesis, and metastasis of malignant tumors. CTSB also plays an important role in implantation, pregnancy, and embryonic development, the dynamic state of transcription and translation of CTSB is coupled with early embryonic development^13^.

**Catalytic function of CTSB depends on pH.** CTSB has an additional pH-sensitive occluding loop that enables it to act as an endopeptidase/exopeptidase depending on pH 14. Lysosomes are compartments surrounded by membrane bilayers with acidic pH, which is optimal for many hydrolytic enzymes. The low pH of lysosomes provides an ideal environment for CTSB activation 15. CTSB normally functions in acidic lysosomes to degrade proteins and maintain cellular homeostasis. However, in many human diseases, CTSB is translocated to the cytosol at a neutral pH, where it activates inflammation and induces cell death. CTSB is active at both neutral pH (7.2) in the cytosol and acidic pH (4.6) within lysosomes 16. CTSB not only has endopeptidase and carboxyl dipeptidase activities but also has noteworthy ligase activities 17. Owing to its dual roles as an endopeptidase and peptidyl dipeptidase, CTSB can participate in the early and late stages of lysosomal protein breakdown 2.

**Localization of CTSB determines its primary function.** CTSB was originally thought to function only within lysosomes, and it is now widely accepted that CTSB exhibits both extra-lysosomal and extracellular functions 18. The localization of CTSB in intracellular lysosomes can lead to the secretion of inactive and active forms, mainly regarded as housekeeping enzymes involved in intracellular protein degradation of extracellular (by endocytosis or phagocytosis) or other intracellular compartments (autophagy) 19. CTSB located in lysosomes can promote autophagy and immune responses by trafficking TNF-α-containing vesicles to the plasma membranes of macrophages 20. CTSB, located in secretory vesicles, the cytoplasm, and the nucleus, can bind to the plasma membrane for secretion into the extracellular space, which is associated with cell surface cavities and specialized membrane microstructural domains involved in signaling pathways, endocytosis, and proteolysis 9. The CTSB located in the mitochondria is thought to trigger cell death 21. Due to the localization of CTSB in the nucleus, where it is associated with nuclear scaffolds, CTSB is thought to be involved in cell division 22. In addition, in some cancer cells, CTSB is localized to the cell surface and secreted into the extracellular environment 23.

**CTSB degrades and remodels the extracellular matrix (ECM).** CTSB can participate in ECM remodeling by degrading structural components (collagen and elastin). ECM remodeling not only allows cell separation and invasion but can also release angiogenic and growth factors that bind to the matrix 24. Additionally, CTSB initiates the proteolytic cascade by activating other tumor-promoting proteases, including matrix metalloproteinases (MMP) and urokinase-type proenzyme activators 25. Under physiological conditions, CTSB plays an important role in the degradation of a variety of proteins. Under pathological conditions, CTSB can hydrolyze a variety of ECM components 26.

**CTSB mediates cell cycle arrest.** As a lysosomal cysteine protease, CTSB can degrade intracellular proteins in the lysosomes. CTSB inhibits cell proliferation in mouse follicular granulosa cells. Knockdown of CTSB increases the proliferation of mouse granulosa cells by increasing the phosphorylation of ERK and AKT in vitro and regulates cell cycle progression by increasing the expression of Myc and cyclin D2 27. However, CTSB knockdown suppressed cancer cell proliferation. In the human cholangiocarcinoma cell line QBC939, CTSB expression inhibited by mir-637 can decrease proliferation ability and promote apoptosis 28. CTSB can colocalize with p27, resulting in the degradation of p27 in lysosomes 29. Gopinath et al. reported that CTSB and urokinase plasminogen activator receptor (uPAR) decreased the expression of the cycle-dependent kinase inhibitor p27. In glioma cells, knockdown of CTSB and uPAR reduced tumor growth and increased p27 nuclear expression in vivo. Increased p27 expression induces G0/G1 arrest and ERK/AKT signaling is involved in G0/G1 arrest 30.

**Figure 1:**
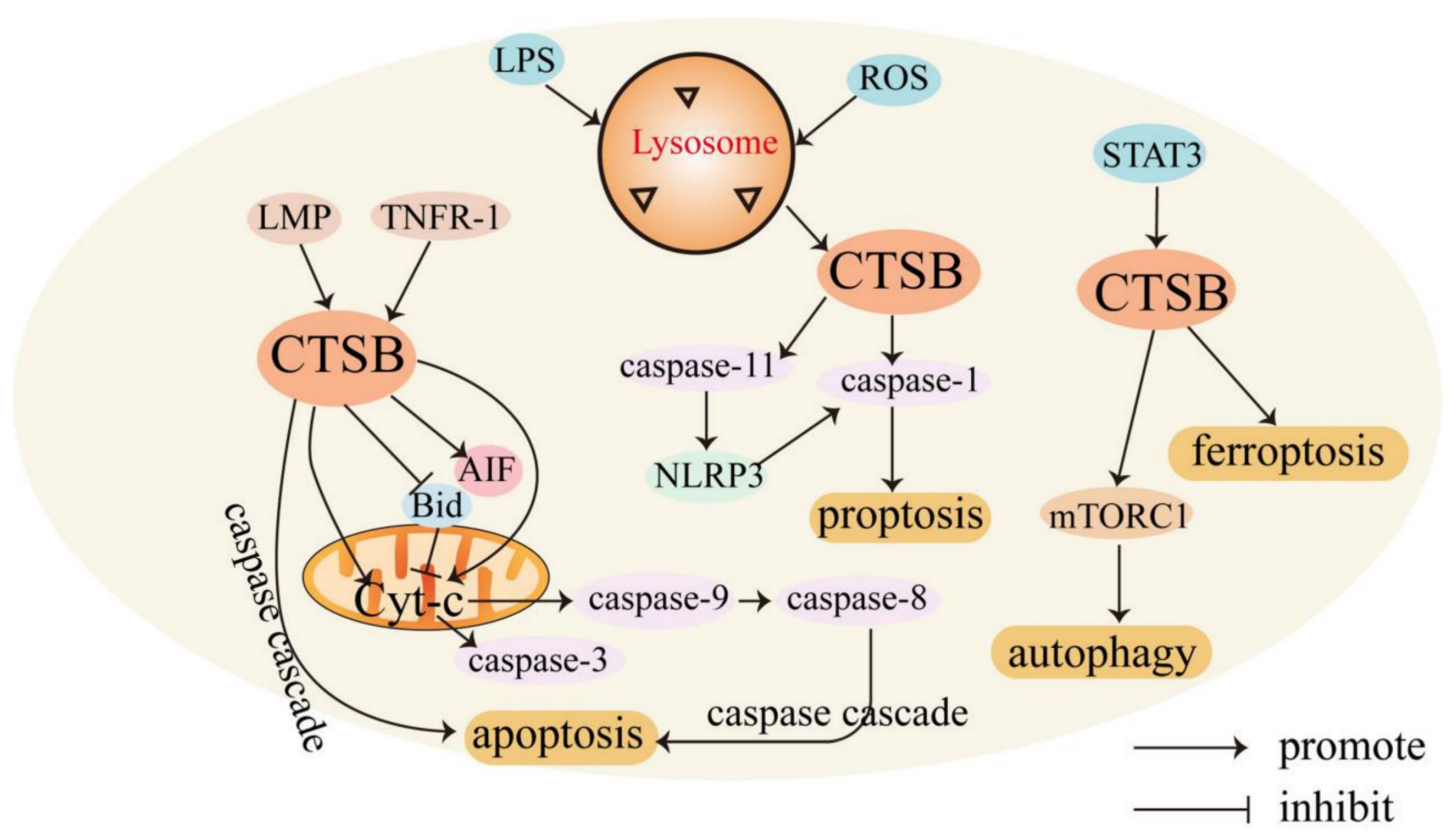
Role of CTSB in cell death. In the presence of LMP, CTSB is released into the cell matrix, leading to lysosome-dependent death, and triggers the activation of the apoptotic cascade by releasing Cyt-C. CTSB also independently leads to apoptosis independent of the caspase pathway. LPS promotes the activation of CTSB and activates caspase-1, leading to pyroptosis. STAT3 promotes ferroptosis by activating CTSB-mediated lysosomal cell death. CTSB contributes to the activation of the mTORC1 used and thus active autophagy.

**CTSB mediates cell death.** CTSB is involved in regulating various forms of cell death. Usually, CTSB is localized in lysosomes and released into the cytoplasm, triggering apoptosis through different pathways. CTSB can cause cell death through lysosomal membrane permeability (LMP) 31. LMP can cause CTSB to translocate to the cytoplasmic matrix, leading to lysosome-dependent death. The regulatory mechanism of CTSB in mediating cell death is shown in Figure 1.

The lysosome-mitochondrial axis of cell death has been proposed to indicate that apoptosis is regulated by CTSB 32. Releasing CTSB from lysosomes can promote the release of cytochrome c (Cyt-C) from the mitochondria, resulting in the cleavage of caspase-9 and caspase-3 and subsequent induction of apoptosis 33. CTSB can also induce the activation of caspases or release pro-apoptotic factors from the mitochondria in response to certain stresses 34. In addition, CTSB cleaves Bid, a pro-apoptotic Bcl-2 family member, and truncated Bid translocates to the mitochondria, induces the release of Cyt-C, and triggers the activation of the apoptotic cascade 35, 36. CTSB may also be involved in TNF-α-triggered apoptosis by releasing mitochondrial Cyt-C, and triggering TNFR-1 to activate small amounts of caspase-8 37. Upon TNF-α stimulation, CTSB acts as an important downstream mediator of the apoptosis cascades triggered by TNF and hemichannase 38.

CTSB can also induce apoptosis independently. In apoptosis studies of the myelodysplastic syndrome-derived cell line P39, apoptosis was mediated by direct caspase activation by CTSB without a decrease in mitochondrial membrane potential or the release of Cyt-C 39. In non-small cell lung cancer, CTSB is essential for caspase-independent cell death induced by paclitaxel, epoxyketone B, and discomulin 40. In a study of the TNF-mediated apoptosis pathway in WEHI-S fibrosarcoma cells, CTSB also induced apoptosis independent of caspase 41. In glioma-initiating cells (GICs), downregulation of uPAR and CTSB caused a transcriptional arrest and sensitized glioma cells to apoptosis by inhibiting c-Met signaling 42. During acute pancreatitis, CTSB leaks from colocalized organelles into the cytoplasmic matrix, and cytosolic CTSB activates the intrinsic apoptotic pathway by cleaving and activating Bax 43. In oral squamous cell carcinoma, CTSB-mediated proteolytic events are required for TRAIL-triggered apoptotic pathways 44. CTSB activation can enhance HDACi- and adriamycin-induced apoptosis in myeloma cells, which may be related to the nuclear translocation of apoptosis-inducing factor (AIF). RNAi-mediated downregulation of uPAR and CTSB has also been found to trigger a partial extrinsic apoptotic cascade accompanied by nuclear translocation of AIF in gliomas 45. Signal transducer and activator of transcription 3 (STAT3) enhances the expression of CTSB and cathepsin L in a transcriptional manner, possibly through the downregulation of mRNA for serine protease inhibitor 2A, an anti-apoptotic cytosolic cathepsin inhibitor that protects lysosomes from CTSB-driven permeabilization 46. STAT3-mediated upregulation of CTSB and CTSL enhances cell death via LMP 47.

CTSB is also involved in regulating necrotic-like programmed cell death, pyroptosis, ferroptosis 48, and autophagy 49. Necrotic-like programmed cell death involves the production of reactive oxygen species (ROS). ROS may be a direct consequence of Bax/Bak-mediated mitochondrial membrane permeability and ROS production induces LMP, resulting in the release of CTSB. CTSB release and caspase activation amplify TNF-α apoptosis signaling 50. Endocytosis of high-mobility group box 1 induces CTSB release from ruptured lysosomes and the formation of pyroptosome and caspase-1 activation, resulting in pyroptosis 51. Cytoplasmic lipopolysaccharide (LPS) promotes the activation of CTSB and subsequently cleaves pro-inflammatory caspase-11 into enzymatically active caspase-11, leading to the activation of the non-canonical NLRP3 inflammasome, which can directly activate caspase-1 and subsequently induce the secretion of pro-inflammatory factors, ultimately leading to pyroptosis 52. CTSB is also a mediator of the organelle-specific triggering of ferroptosis, an iron-dependent non-apoptotic cell death driven by excessive lipid peroxidation and subsequent membrane damage 53. CTSB mediates ferroptosis by activating DNA damage-induced autophagy 54. Transcriptional upregulation of CTSB in ferroptosis requires the activation of STAT3. STAT3 promotes ferroptosis by activating CTSB-mediated lysosomal cell death 55.

Autophagy is an intracellular degradation system involving lysosomes that engulf cytosolic components by forming double-membrane vesicles (autophagosomes) and degrading these engulfed components using lysosomal enzymes 56. CTSB exerts dual effects on autophagy regulation. Accumulation of autophagy markers (LC3-II) has shown that CTSB gene deletion or drug inhibition can lead to lysosomal biogenesis and autophagosome formation 57. However, in mouse follicular granulosa cells, knockdown of CTSB significantly decreased the mRNA expression of the autophagy marker genes LC3-1 and ATG5 27. In pancreatic beta cell (INS-1) cells treated with 400 μM mono (2-ethylhexyl) phthalate, CTSB contributed to the activation of mTORC1 to inhibit autophage 58.

## CTSB in non-tumor diseases

**The role of CTSB in Alzheimer's disease.** The role of the CTSB in brain function has been debated. CTSB is involved in cell death following brain injury and is neuroprotective. In humans, plasma levels of CTSB are positively associated with health and memory and can be increased by physical exercise 59. Accumulation of amyloid-β (Aβ) has been implicated as a critical trigger for Alzheimer 's disease (AD). CTSB degrades Aβ by truncating Aβ1-42 at the C-terminus, protecting nerves by reducing Aβ levels, and improving neuronal dysfunction. Low CTSB activity may promote AD, and increased CTSB activity may counteract the neuropathology of this disease and provide therapeutic strategies 60. However, CTSBs can generate pyroglutamic acid amyloid beta peptide (pGlu-Aβ), a particularly deleterious form of Aβ present in AD. The PGlu-Aβ peptide is more stable and neurotoxic and leads to increased Aβ aggregation 61. CTSB may also cleave amyloid precursor protein and exacerbate neuronal defects in AD 62. CTSB may act as a secretase to participate in the regulated secretory pathway of brain neurons by degrading Aβ to protect the brain neurons and produce PGlu-Aβ to aggravate AD lesions.

**CTSB involved in the cardiovascular disorders by degrading ECM.** CTSB is widely expressed in cardiac muscle 63, plays a central role in ECM remodeling, and is associated with the development and progression of cardiovascular diseases. ECM proteins, including elastin, laminin, and collagen (types I and III), constitute normal cardiac structures 64. The altered expression of several ECM-degrading proteases (e.g., MMPs and serine and cysteine proteases) affects cardiac remodeling, which is an important potential cause of several cardiovascular diseases 65. CTSB is involved in the pathophysiological processes of cardiovascular-related diseases, including atherosclerosis, myocardial infarction, hypertension, myocarditis, chemotherapy-induced myocardial injury, and heart failure. As indicators of autophagic activity, CTSB and cathepsin D are involved in the regulation of cell death and survival during the development of atherosclerosis 66. CTSB plays various roles in the evolution of extracellular and intracellular atherosclerotic plaques. CTSB secreted by macrophages can post-translationally modify apoA-I by cleaving its C-terminal Ser228, thereby greatly reducing its lipid solubility, decreasing the ability of low-density lipoproteins to export cholesterol, and exacerbating the disease process of atherosclerosis 67. CTSB may also be involved in the degradation of myogenic fibronectin in myocardial infarction. Circulating levels of CTSB were found to be higher in patients with acute myocardial infarction than in controls 68. Inhibition of CTSB affects the development of hypertension. As a type of αENaC cleaving enzyme, blockade of CTSB can prevent hypertension 69. In addition, CTSB is associated with blood pressure and may be involved in aortic remodeling 70. CTSB can aggravate coxsackievirus B3-induced myocarditis by activating inflammasomes to promote pyroptosis 71. CTSB is also involved in stress-induced cardiomyocyte apoptosis, regulation of cardiac hypertrophy, and remodeling via the TNF-α/ASK1/JNK pathway 72.

**CTSB can active autophagy in obesity.** Obesity promotes the hypertrophy and hyperplasia of adipocyte, and weakens the regulation of fat storage in white adipose tissue, which results in impaired lysosomal function and the release of CTSB and other proteases into the cytoplasm, leading to autophagosome accumulation and increased inflammation. Activation of CTSB induces mitochondrial dysfunction, increases ROS production, and releases mitochondrial proteins (such as Cyt-C), which cause adipocyte death 73. With the development of obesity, white adipose tissue secretes pro-inflammatory cytokines (IL-6 and TNF), leading to chronic low-grade inflammation 74. Among the lysosomal proteins, CTSB is the most abundant protease required to activate autophagy. In obesity, autophagy activation relieves metabolic stress and inflammation and restores cellular homeostasis 75. CTSB can promote lipogenesis by degrading the fibronectin network and inhibiting Wnt/β-catenin signaling 76.

**CTSB is assocated with lung disorder by degrading ECM.** CTSB induced emphysema in an experimental model of emphysema and cathepsin release in response to cigarette smoke. CTSB release in smoking-related lung diseases leads to ECM degradation and emphysema 77. Extracellular neutrophil elastase (NE) can activate CTSB 78 and increase its expression. CTSB released by NE stimulation may lead to ECM degradation, produce emphysema commonly seen in lung disease, and affect the function of important antimicrobial proteins and peptides 79. NE can induce interleukin (IL)-8 expression in human bronchial epithelial cells via the NF-κB activation pathway 80. NF-κB mediates CTSB and MMP-2 activation in human bronchial epithelial cells after doxorubicin and LPS treatment. NF-κB pathway inhibited by SN50 results in decreased expression of CTSB and MMP-2, and NE has been shown to induce IL-8, CTSB, and MMP-2 production via the interleukin-1 receptor associated kinase-1/Toll-like receptor-4-mediated macrophage pathway 79, which leads to degradation of ECM, followed by emphysema common in lung diseases.

## The role of CTSB in cancer

CTSB has been described as a “multifunctional” enzyme in cancer 81. CTSB plays a dual role in tumor malignant progression, and the entire process depends on a delicate balance between proteases and their inhibitors as well as between pro-apoptotic and pro-invasive properties. As a prognostic marker and potential therapeutic target in various types of cancer, CTSB is one of the most potent tumor-promoting cysteine cathepsins, overexpressed in various human cancers and secreted by malignant cells and cells of the tumor microenvironment 82. CTSB is involved in a plethora of malignant progression processes, including tumor growth, angiogenesis, invasion, and metastasis.

**CTSB and tumor angiogenesis.** The development of new blood vessels in tumors depends on the production of vascular endothelial growth factor (VEGF) released by tumor cells and/or matrix cells. Proteolytic remodeling of the ECM is a key event in vascular sprouting during angiogenesis 83. CTSB promotes ECM remodeling to facilitate neovascularization. However, it is unclear whether CTSB is pro- or anti-angiogenic. In endothelial cells (but not in tumors), CTSB can inhibit angiogenic responses. CTSB maintains endothelial cells in a non-angiogenic state by increasing endothelial statin production while decreasing VEGF expression, suggesting that CTSB may act as a suppressor of angiogenesis at the endothelial level. It has been reported that overexpressed CTSB interferes with the angiogenic process by downregulating VEGF and upregulating the angiogenic inhibitor endostatin in bovine retinal ECs 84. In pathological situations, CTSB promotes angiogenesis when it acts on tumor cells or the ECM. CTSB inhibition delayed angiogenesis and tumorigenesis in vivo 85. Recently, it was shown that the knockdown of uPAR and CTSB can inhibit tumor-induced angiogenesis by disrupting the JAK/STAT pathway-dependent expression of VEGF 86. Similarly, another study showed that inhibition of CTSB expression can inhibit glioblastoma (GBM)-induced neovascularization 87. Elevated CTSB activity, which results in increased secretion or mobilization of VEGF, enhances the induction of tumor angiogenesis 88. In GBM, CTSB is mainly located at the invasive margin of tumor infiltration and neovascularization 89. Degradation of tenascin-C around new blood vessels by CTSB can promote the expansion of new blood vessels, leading to glioma progression 90. CTSB promotes angiogenesis by degrading inhibitors of MMPs (TIMPs) 91 or by releasing growth factors, such as VEGF and transforming growth factor (TGF)-β 92.

**CTSB acts on the tumor microenvironment.** Tumor microenvironment (TME) is the cellular environment in which cancer or CSCs exist 93, and is composed of tumor cells, tumor stromal cells, and acellular components of the ECM 94. Cathepsins are usually highly expressed in various types of immune cells and fibroblasts 95. Many tumor microenvironments are acidified, which may be associated with CTSB, as this lysosomal enzyme is optimally active at a slightly acidic pH 96. The environment of acidic tumor cells also favors the release of CTSB 97. Many proteases have been implicated in pathogenic processes that occur in the co-evolution of cancer cells and their microenvironment, including MMPs, urokinase-type plasminogen activators, and cathepsins 98. Proteases capable of degrading ECM proteins have recently been identified as critical regulators of the TME, and secreted CTSBs significantly alter the TME during invasion. However, their mechanism of action and associated intracellular signaling pathways have not been identified 99, and CTSBs of stromal cells from tumors or microenvironments play critical roles in multiple stages of tumor growth and metastasis 100. Previous studies have indicated that cysteine proteases are compromised by the microenvironment 101. Moreover, the TME can regulate CTSB expression in tumor cells and other tumor-associated cell types (including stromal fibroblasts and inflammatory cells), and stromal cells of the TME are the main source of CTSB expression 102.

**Figure 2:**
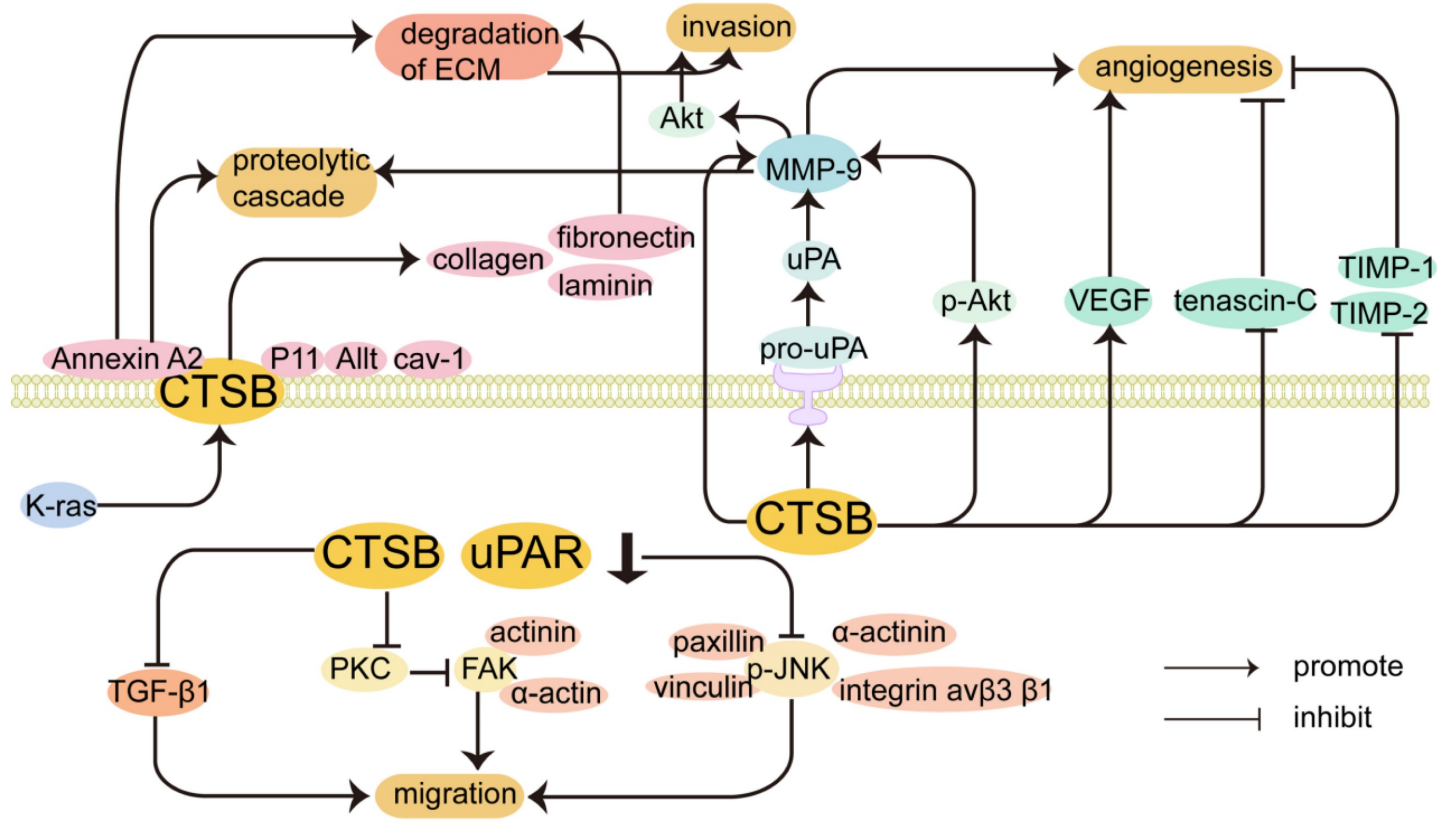
Inhibition of CTSB expression can inhibit migration by affecting other proteins and signaling pathways. CTSB is translocated to the cell surface and participates in the proteolytic cascade, leading to ECM degradation. It interacts with related proteins on the cell surface to promote tumor invasion and metastasis. The expression of CTSB also plays a role in tumor angiogenesis.

**CTSB promotes the invasion and migration of cancer cells.** Release of proteolytic enzymes that cause degradation of the ECM, damage to the basement membrane, and migration of cancer cells in response to chemokines. Increased CTSB expression occurs at the invasive margin in many tumors, and degradation of the ECM by CTSB is a critical step in cancer cell invasion and metastasis 29. The regulatory mechanism by which CTSB promotes the invasion and migration of cancer cells is shown in Figure 2. CTSB promotes tumor invasion and metastasis by degrading the ECM, which is mediated by a proteolytic cascade. Biochemical studies have identified that collagen I, collagen IV, fibronectin, and laminin can be substrates of CTSB, and CTSB can proteolytically degrade these ECM components and drive tumor cell invasion 103. Elevated levels of CTSB alone or in combination with other proteolytic enzyme pathways have also been associated with tumor progression 8. In cancer cells, CTSB is shuttled to the plasma membrane, where it activates receptor-bound urokinase-type plasminogen activator (pro-uPA). uPA activates plasminogen, a serine protease that digests ECM proteins and activates MMPs to degrade the ECM 104. It has been found that the CTSB can inactivate TIMP-1 and TIMP-2. CTSB promotes cell invasion by disrupting inhibitors to enhance MMP activity and inactivate TIMP-1 and TIMP-2. CTSB can upregulate the expression of MMP-9 by activating the PI3K/Akt pathway, and the expression of p-Akt was significantly increased in CTSB-overexpressing cells, indicating that CTSB upregulation can enhance Akt signaling, which in turn leads to the upregulation of MMP-9, which may also lead to the generation of proteolytic cascades 105. In hepatocellular carcinoma, the phosphorylation of Akt in CTSB-overexpressing cells was significantly increased, which may also indicate that CTSB regulates the progression of HCC through the PI3K/Akt signaling pathway. In addition, interactions between integrins and extracellular components play an important role in tumor differentiation and progression. Inhibiting integrin αvβ3 significantly prevented the CTSB-overexpressing phosphorylation of Akt, which can inhibit cell proliferation and lead to cell death. Collectively, the CTSB/integrin αvβ3/PI3K/Akt axis is critical for the regulation of HCC progression 106. Knockdown of CTSB may inhibit HL-60 cell proliferation and tumorigenesis through inactivation of the Akt signaling pathway *in vitro* and *in vivo* 107.

Caveolae (Cav) are cell surface binding sites for CTSB 9, and proteases bound to the surface of tumor cells and secreted by tumor cells and tumor-associated host cells promote local protein hydrolysis during tumor invasion 108. In inflammatory breast cancer cells, overexpression of caveolin-1 contributes to the proteolytic cascade involving CTSB, leading to ECM degradation 109, 110. Caveolin-1, the main structural protein of Cav, is associated with ECM degradation and invadopodia formation 111. In malignant human colorectal carcinoma cells, CTSBs are attached to the plasma membrane at specific micro-regions, invadopodia, and Cav 112. The binding of CTSB to Cav is regulated by the activation of K-ras, which increases active CTSB trafficking to the cell surface with positive invading ability 113.

CTSB may also be involved in the metastatic colonization of tumors via annexin A2, and the cellular matrix is crucial for the metastatic colonization of tumor cells in distant organs 114. CTSB can also bind annexin A2 on the cell surface 115 and is involved in the activation of binding proteins in discrete regions of the extracellular space, which may explain the role of CTSB in tumor invasion and metastasis. CTSB co-localizes with the cell surface protein annexin A2 to promote invasiveness and angiogenesis in GBM 116. The protein complex annexin A2-S100A10 heterotetramer (AIIt) can act as a binding protein for CTSB on the tumor cell surface and localizes on the extracellular surface, where it enhances the activation of proteases. S100A10 can lead to the activation of pro-CTSB. The light chain of AIIt, p11, also serves as a binding site for CTSB on the surface of tumor cells, and CTSB binds to the plasma membrane through its interaction with p11 117. In conclusion, the co-localization of proteases and their substrates on the surface of tumor cells may contribute to (1) activation of protease precursor forms and initiation of protein hydrolysis cascades and (2) selective degradation of extracellular matrix proteins 118. In the colorectal cancer HCT116 cell line, the increased trafficking of CTSB and uPA to the Cav of cells correlates with the expression of p11 in the cavernous body of cells, which indicates that p11 may be a binding partner of CTSB in the Cav to increase the interaction between CTSB and uPA/uPAR, thereby triggering the proteolytic cascade 113. In the lung adenocarcinoma cell line A549, the downregulation of annexin A2 expression can inhibit the secretion of MMP-2 and CTSB. Annexin A2 also promotes lung cancer progression by regulating the expression of CTSB and MMP-2 119. The absence of CTSB impaired the development of high-grade invasive ductal carcinomas and reduced the metastatic burden in the lungs 120. CTSB secreted by macrophages enhances the metastasis of breast cancer cells to the lungs 100. It has been shown that CTSB secreted by fibroblasts induces elevated expression of stearoyl-CoA desaturase 1 in B16F10 cells through annexin A2 and increased stearoyl-CoA desaturase 1 expression ultimately affects cancer cell proliferation and metastatic colonization. Fibroblast-secreted CTSB mediated stearoyl-CoA desaturase 1-induced fatty acid composition changes in fatty acid composition in tumor cells, thus determining the initiation of lung metastasis of melanoma 121.

CTSB promotes tumor progression not only by proteolytic function but also by a series of signal transduction pathways. CTSB is one of the major intracellular interaction partners of hepatitis B splice protein, which was identified using a yeast two-hybrid screening assay 122. The interaction between hepatitis B spliced protein and CTSB may promote the motility and invasion of HCC cells 123. Metastasis-associated protein 1, which inhibits the expression of CTSB and inversely correlates with E-cadherin, is essential for bone metastasis 124. E-cadherin is a substrate of cathepsin, and overexpression of CTSB correlates with the downregulation of E-cadherin.

In gliomas, both uPAR and CTSB are overexpressed and can degrade the ECM alone or in combination 45. Studies have shown that CTSB and uPAR synergistically regulate the migration of GICs and are closely related to the occurrence and development of gliomas 45. It has been reported that blocking uPAR and CTSB expression results in a significant reduction in inflammatory breast cancer cell migration and invasion 108. shRNA-mediated downregulation of uPAR and CTSB downregulates focal adhesion kinase (FAK) binding to α-actin and actinin in vitro by disrupting the PKC-integrin complex. Blocking the radiation-induced interaction between FAK and cytoskeletal molecules can induce cytoskeletal disassembly in GICs and non-GICs 125. Downregulation of CTSB induces cytoskeletal disorganization, reduces cell motility, and inhibits tumor cell migration. In the JNK-MAPK signal transduction pathway, CTSB promotes the migratory activity of GIC by phosphorylating JNK (p-JNK), and JNK activation is required for cell migration. Cytoplasmic p-JNK interacts with adhesion molecules (integrin av β3β1) and motility molecules (paxillin, vinculin, and α-actinin) to drive cells toward an invasive and migratory phenotype. Reducing cytosolic p-JNK by downregulating uPAR and CTSB also induces the translocation of MEKK-1-P-JNK complexes from the cytoplasmic matrix to the nucleus, which can inhibit the migration of glioma cells. Moreover, uPAR and CTSB regulate JNK activation and translocation via the Ras-Pak-1 pathway.

Increasing evidence has shown that many human cancer cells maintain intercellular adhesion and invade as cohesive multicellular groups, which is called collective cell invasion or migration 99. CTSB is important for collective invasion because it can break down ECM components and regulate proteolytic networks and signaling pathways. Degradation of the basement membrane and connective tissue by various proteases is considered a prerequisite for the collective invasion of cancer cells. FAK can regulate collective cell invasion by regulating cell-cell adhesion via controlling E-cadherin internalization 126. Knockdown of CTSB in salivary adenoid cystic carcinoma-83 (SACC-83) cells inhibits FAK expression 127, induces cytoskeletal disorganization, inhibits ECM remodeling, and impairs the formation of leader cells 125. CTSB can be used to define leader cells in SACC 127. CD147 promotes extracellular hydrolysis and lysosomal collagen degradation in the ECM by upregulating the expression and activation of CTSB in human HCC. CTSB knockdown and CA074 treatment inhibited 3D collective invasion, whereas upregulation of CTSB promoted cell migration and collective invasion in HCC. CTSB overexpression makes polyoma middle T cancers more aggressive by increasing proteolytic ECM protein degradation and promoting collective cell invasion into adjacent tissues 128.

**CTSB interacts with TGF-β1 to promote tumor invasion.** Transforming growth factor-β (TGF-β) is involved in some carcinogenic effects during advanced tumorigenesis through MAPK, JNK, p38, and PI3K pathways 129. In the melanoma cell line WM793, CTSB activity is important for TGF-β production and secretion 130. TGF-β1 induces the invasion of malignant meningioma cells with associated upregulation of uPA, CTSB, and MMP-9, and uPAR and CTSB act upstream in TGF-β1-initiated signaling 131. In myeloid tumors, alterations in the TGF-β1 signaling pathway lead to upregulation of CTSB, and TGF-β1 stimulates the expression of CTSB mainly by inhibiting Smad1 132.

**Epithelial-mesenchymal transition (EMT) and CTSB.** ECM remodeling is an important process that enhances cell invasiveness during EMT, involving increased production of ECM-degrading peptidases 133. The E-box at nucleotides 7 to 2 of the CTSB promoter is essential for CTSB expression, and the binding of upstream stimulatory factor 1 and upstream stimulatory factor 2 to this E-box can modulate CTSB promoter activity 134. The EMT activator zinc finger E-box binding homeobox 1 is a key promoter of metastasis and stemness 135. Regulation of CTSB promoter activity through E-box elements associated with EMT activators.

TGF-β is an important activator of EMT, and CTSB can also directly release and activate TGF-β1 via proteolytic ECM components 92, 136, 137. The role of CTSB in the TGF-β1 signaling pathway is also a key signal that triggers EMT in cancer. E-cadherin serves as a hallmark marker of the epithelial cell phenotype, and the overexpression of CTSB and cathepsin X decreases the expression of E-cadherin. Vimentin is considered a marker of the mesenchymal phenotype, and silencing CTSB and cathepsin X reduces vimentin expression 136.

CTSB also upregulates EMT-activated transcription factors through the Wnt/β-catenin pathway 12. In thyroid cancer, CTSB localizes to the basement membrane and induces EMT by altering the ECM. CTSB overexpression induced cell migration by enhancing vimentin and Snail expression in thyroid cancer cell lines. Changes in CTSB expression or secretion during thyroid cancer regulate metastasis by activating p38-mediated EMT 138. After TGF-β-mediated EMT, CTSB proteolyzed Deactented-2 after continuous treatment with TGF-β, resulting in loss of the mesenchymal phenotype 139. In the colon adenocarcinoma HT29 cell line, CTSB was upregulated in Snail-overexpressing cells and accumulated in the invasive front. In snail-overexpressing HT-29 cells, CTSB activity and invadosome localization are associated with the mesenchymal phenotype in colon cancer 140.

**CTSB and cancer stem cells.** Cysteine cathepsins play a key role in regulating CSCs function, and CTSB is highly expressed in GBM stem cells. In GIC, simultaneous knockdown of uPAR and CTSB significantly reduced the expression of stemness markers and resulted in a reduction in the size and number of GIC spheres 141. CTSB is expressed in two CSC subsets of moderately differentiated oral tongue cell carcinoma, in which it has been demonstrated to localize to CSC within the tumor nest and stroma surrounding the tumor 142. CTSB has also been associated with prostate CSC. In the presence of adipocytes, prostate cancer cells actively secrete cholecystokinin (CCK), which stimulates the self-renewal of prostate CSCs and induces CTSB production in adipocytes. In return, CTSB promotes the secretion of CCK by cancer cells. More importantly, inactivation of the CCK receptors not only inhibits CTSB secretion from adipocytes but also synergizes the inhibitory effect of CTSB inhibitors on adipocyte-promoted self-renewal of prostate CSCs 143. CTSB is also expressed in CSCs in head and neck cutaneous squamous carcinoma 144 and pancreatic cancer stem-like cells. CTSB is expressed on the surface of pancreatic cancer stem-like cells and directly or indirectly affects the extracellular microenvironment 145. We previously reported that cobalt chloride or chemoradiotherapy induced the formation of polyploid giant tumor cells (PGCCs) 146. PGCCs have CSC properties and can express the CSC markers CD44 and CD133 147. Daughter cells generated by PGCCs via asymmetric cell division exhibit strong migration, invasion, and proliferation abilities. CTSB is significantly elevated in PGCC and daughter cells 148.

## Clinical practice of CTSB

**CTSB and drug resistance.** CTSB plays an important role in controlling the balance between apoptosis and necrotic death under stress conditions 149. Drug-resistant human laryngeal carcinoma cells may exhibit high levels of CTSB 150. The conjugation of the second mitochondria-derived activator of caspases and CTST-cleavable peptide to doxorubicin can specifically cleave to pro-apoptotic second mitochondria-derived activator of caspases and cytotoxic doxorubicin in CTSB-overexpressing cancer cells, inducing a synergy of the pro-apoptotic activity with chemotherapy 151. In the highly chemoresistant osteosarcoma Saos-2 cell line, palladacycle concentrates in lysosomes to induce LMP, favoring CTSB release into the cytoplasmic matrix to induce cell death 152. In the treatment of relapsed and refractory multiple myeloma, the anti-CD38 monoclonal antibody SAR650984 effectively killed resistant multiple myeloma cells by increasing CTSB throughout the cytoplasm 153. In chronic myelogenous leukemia CD34^+^ cells, imatinib induced CTSB activation, and overexpression of CTSB sensitized these cells to imatinib killing 154. Salinomycin and gefitinib synergistically induce apoptosis via CTSB and CTSD, triggering mitochondrial lysosomal crosstalk and caspase-independent pathways in colorectal cancer cells, and this new combination therapy may provide potential clinical applications to overcome GEF resistance in colorectal cancer 155.

CTSB is a direct target gene of miR-140, and overexpression of miR-140 reduces CTSB levels, enhances temozolomide cytotoxicity, inhibits mesenchymal transition, and affects CTSB-regulated tumor spheroid formation and expression of stemness markers. In GBM, high expression of CTSB is related to the intrinsic drug resistance of TMZ. The mechanism of resistance may involve upregulation of CTSB, reversing miR-140-suppressed mesenchymal transition and sensitizing TMZ to GBM cytotoxicity 156. In germinomas, monanchocidin A increases the activity of cisplatin in germ cell tumor cells. Cisplatin and monanchocidin A have a synergistic effect in NCCIT-R cells (cisplatin-resistant germ cell tumor cells), and high concentrations of MonA can induce LMP, which is released into the ECM, resulting in the non-selective degradation of cellular components. MonA may overcome resistance to currently applied therapies through autophagy and apoptosis caused by the release of CTSB into the ECM due to LMP 157. CTSB-mediated scramblase activation triggers cytotoxicity and cell cycle arrest by andrographolide to overcome cellular resistance in cisplatin-resistant human hepatocellular carcinoma HepG2 cells 158.

**CTSB and cytotoxic drugs.** Prodrugs are pharmacologically inactive forms of active drugs 159, and may be an effective way to selectively release active drugs through high cathepsin expression in the tumor microenvironment, improving the safety of chemotherapy with fewer toxic side effects 160. CTSB has been shown to be an effective target for doxorubicin delivery. CTSB exhibits enzymatic activity, which increases the chemotherapy potential of doxorubicin and limits its toxicity. The CTSB-sensitive precursor drug concept has been used for targeted release 161. As a precursor drug-activating enzyme, CTST can cleave doxorubicin precursor drugs. doxorubicin prodrugs remain inactive in vascular and normal tissues, and free doxorubicin cleaved by CTSB can be released into cancer cells, leading to targeted cytotoxicity. CTSB cleaves the doxorubicin prodrug to release the free drug in the presence of CTSB and in a subacidic environment. Studies have shown that CTSB-cleavable doxorubicin prodrugs are less toxic *in vitro* and more effective *in vivo*, demonstrating the effect of CTSB 161. PNP (CTSB-specific doxorubicin prodrug nanoparticles) selectively released free doxorubicin only in CTSB-overexpressing peritoneal carcinoma cells, thus greatly reducing local and systemic toxicity. PNP specifically releases free doxorubicin into the nucleus of cancer cells via the sequential enzymatic cleavage of CTSB and intracellular proteases. However, owing to the innate low CTSB expression in normal cells, most PNP remain inactive in the cytoplasm of normal cells, and cytotoxicity to CTSB-deficient normal cells is greatly reduced 162. CTSB-overexpressing tumor cells activate the albumin-bound doxorubicin prodrug Al-ProD (CTSB cleavable peptide (FRRG) and doxorubicin) for targeted cancer therapy. Doxorubicin is selectively released in CTSB-overexpressing tumor cells to induce effective antitumor efficacy. Simultaneously, Al-ProD increases the safety of chemotherapy by maintaining an inactive state with significantly reduced toxicity in normal tissues with inherently low CTSB expression 160. CTSB can completely or partially prevent the toxicity of HDAC, ERBB1/2/4 inhibitors, nalatinib, nilotinib, acid keratamide, thymquinone, and tyrosine kinase inhibitors, such as sunitinib and pazopanib 11.

## The inhibitors targeting CTSB

Given the multiple roles of CTSB in tumors, several CTSB inhibitors have been developed and studied to inhibit tumor invasion in different cancer treatments, although their clinical effect has not been demonstrated 163. Proteases and their endogenous inhibitors have been shown to have prognostic value in characterizing tumor aggressiveness and disease outcomes 164. CTSB is one of the most important lysosomal cysteine proteases with the highest activity in acidic environments. In addition to ambient pH, CTSB activity is primarily influenced by its inhibitors. CTSB and its endogenous inhibitors may promote proteolysis in hepatoma cells, thereby contributing to the invasive phenotype of this cancer. The proteolytic activity may be a potential therapeutic target. Studies have also shown that CTSB inhibitors combined with conventional therapy may have clinical value 165. Proteiase inhibitors have been successfully used in the treatment of HIV and hepatitis C. The current study provides evidence that CA-074 may be a novel therapeutic candidate for the treatment of Multiple sclerosis 166. Studies have also shown that the commonly used chemotherapy doxorubicin directly binds to CTSB to inhibit its activity, demonstrating for the first time the utility of CTSB as a potential therapeutic target for acute myeloid leukemia 167.The demonstration that a clinically viable protocol using E64d is efficacious in TBI animal models enables the development of E64d for traumatic brain injury treatment 168.

**Small-molecule inhibitors and CTSB.** CA-074 is potent selective irreversible CTSB inhibitor, while CA-074Me is its methylated form that increases cell permeability, where still CA-074 is the active form that inhibits CTSB. CA-074Me decreased CTSB activity by altering its active site. CA-074Me is an exogenous and membrane-permeable CTSB inhibitor, which is more cell-permeable and converted to CA-074 by intracellular esterase 169. The sulfur anion in the CTSB structure attacks the carbon of oxirane in the middle ring of CA-074Me, which leads to a change in the active site of the CTSB enzyme and inhibits CTSB activity. In pancreatitis, activated CTSB expression was significantly lower after treatment with CA-074Me, which greatly reduced the incidence of acute pancreatitis 170. CA-074Me has also been reported to decrease TNF-α expression and inhibit apoptosis induced by coxsackievirus B1 in guinea pigs with polymyositis 171. CA-074Me inhibits apoptosis and liver injury in fulminant liver failure 172. Downregulation of CTSB by CA-074 inhibits breast cancer neovascularization and bone metastases 173.

**Endogenous inhibitors and CTSB.** Cystatins decrease CTSB activity. Cystatins are endogenous and reversible inhibitors of cysteine peptidases and are important players in cancer progression. Cystatins primarily act as inhibitors of cysteine cathepsin. The cystatin family consists of three subfamilies: type I cystatin (stefin family, intracellular inhibitors), type II cystatin (cystatin family, extracellular inhibitors), and type III cystatin (kininogen family) 174. Stefin A can form complexes with CTSB and inhibit CTSBs via direct but weak interactions or conformational changes between stefin A and CTSBs, resulting in strong inhibition. Stefins A and B inhibit cathepsin activity and play a role in cancer progression. The negative correlation between stefin A and CTSB expression is associated with tumor malignancy and metastasis 175. Changes in the activity of CTSB and the endogenous inhibitor stefin A are associated with tumor progression and prognosis and can be used as prognostic factors in cancer patients. It has been shown that stefin A produced by myoepithelial cells inhibits early tumor invasion in breast cancer by inhibiting CTSB 176. In addition, overexpression of stefin A can inhibit the migration, invasion, and proliferation of laryngeal cancer cells via CTSB downregulation 177. In human esophageal squamous cell carcinoma cells, the combination of stefin A and CA-074Me further reduced the activity of CTSB 178. In addition, stefin A not only acts by decreasing the activity of tissue proteins but can also be mediated by other mechanisms. Stefin A inhibits TGF-β-induced EMT in lung cancer cells by inhibiting the ERK/MAPK signaling pathway 179.

Cystatin C is an endogenous inhibitor of papain-like cysteine and a natural inhibitor of CTSB. An imbalance between CTSB and cystatin C regulation is critical for the degradation of ECM components, leading to various phenomena, such as tumor metastasis 180. Cystatin C inhibits both the activity and synthesis of CTSB. CTSB has an occluding loop comprising residues 104-126, and blockade of the active site modulates the substrate and inhibitor-binding properties of the enzyme 181. The reduced binding affinity between CTSB and cystatin C may be caused by changes in glycosylation, the presence of cathepsin activators, or the binding of cathepsins to glycosaminoglycans 182. The ratio of CTSB/cystatin C in the serum is clinically significant in the prognosis of cancer patients. The CTSB/cystatin C ratio is significantly higher in patients with cholangiocarcinoma than in healthy groups 183. In esophageal cancer, the cystatin C /CTSB ratio was significantly lower in the esophageal cancer group than in the control group and significantly correlated with the T stage and lymph node metastasis 184. Cystatin C significantly inhibited SAHA-induced CTSB expression in breast cancer cells 185. Cystatin C expression was lower in breast cancer tissues than in non-cancerous tissues, and cystatin C expression is significantly lower in cancerous tissues than in CTSB 186. Cystatin SN (CST1) is a salivary cystatin that forms a tight equimolar complex with cysteine proteases such as cathepsins by binding to their active sites. Heterodimer binding between CST1 and cystatin C was also found to reduce the proteolytic activity and cell invasiveness of CTSB. However, in colon cancer, upregulation of CST1 neutralizes the inhibition of CTSB proteolytic activity by cystatin C and contributes to colorectal carcinogenesis 187. It has also been shown that CST1 impacts cellular senescence during cancer progression. CST1 knockdown decreases the activity of extracellular CTSB, which induces increased inhibitory phosphorylation of glycogen synthase kinase 3β (GSK3β), leading to increased glycogen accumulation and cellular senescence 188.

In conclusion, CTSB is widely involved in the regulation of human malignant tumors and plays an important role in tumor invasion and metastasis, angiogenesis, and cell death. We reviewed the structure and function of CTSB and summarized its expression in different types of tumors and the regulatory mechanisms involved. In addition, the potential role of CTSB in non-neoplastic diseases was discussed. Small molecular inhibitors and microRNA targeting CTSB have been developed to explore the possibility of clinical application. CTSB, a prodrug-activating enzyme, can cleave doxorubicin prodrugs with less toxicity in vitro and is more effective in vivo. At present, the study of prodrugs is in the early preclinical stage, and clinical evaluation of prodrugs should be further strengthened.

## Abbreviations

AD: Alzheimer's disease
AIF: apoptosis-inducing factor
AIIt: Annexin A2-S100A10 heterotetramer
Aβ: amyloid-β
Cav: Caveolae
CCK: cholecystokinin
CSCs: cancer stem cells
CST1: cystatin SN
CTSB: cathepsin B
Cyt-C: cytochrome c
E-box: enhancer box
ECM: extracellular matrix
EMT: epithelial-mesenchymal transition
FAK: focal adhesion kinase
GBM: glioblastoma
GICs: glioma-initiating cells
LMP: lysosomal membrane permeability
MMP: matrix metalloproteinase
LPS: lipopolysaccharide
NE: neutrophil elastase
PGCCs: polyploid giant tumor cells
pGlu-Aβ: pyroglutamic acid amyloid beta peptide
ROS: reactive oxygen species
STAT3: Signal transducer and activator of transcription 3
TGF-β: transforming growth factor-β
TME: tumor microenvironment
uPA: urokinase plasminogen activator
uPAR: urokinase plasminogen activator receptor
VEGF: vascular endothelial growth factor

## References

[1] Greenbaum LM, Fruton JS (1957). Purification and properties of beef spleen cathepsin B. J Biol Chem.

[2] Mort JS, Buttle DJ (1997). Cathepsin B. Int J Biochem Cell Biol.

[3] Howie AJ, Burnett D, Crocker J (1985). The distribution of cathepsin B in human tissues. J Pathol.

[4] Roberts R (2005). Lysosomal cysteine proteases: Structure, function and inhibition of cathepsins. Drug News & Perspectives.

[5] Chen X, Ren X, Zhu Y, Fan Z, Zhang L, Liu Z (2021). Cathepsin B-Activated Fluorescent and Photoacoustic Imaging of Tumor. Anal Chem.

[6] Cavallo-Medved D, Moin K, Sloane B (2011). Cathepsin B: Basis Sequence: Mouse. AFCS Nat Mol Pages. 2011.

[7] Fong D, Chan MM, Hsieh WT, Menninger JC, Ward DC (1992). Confirmation of the human cathepsin B gene (CTSB) assignment to chromosome 8. Hum Genet.

[8] Aggarwal N, Sloane BF (2014). Cathepsin B: multiple roles in cancer. Proteomics Clin Appl.

[9] Cavallo-Medved D, Sloane BF (2003). Cell-surface cathepsin B: understanding its functional significance. Curr Top Dev Biol.

[10] Sentandreu MA, Aubry L, Ouali A (2003). Purification of bovine cathepsin B: proteomic characterization of the different forms and production of specific antibodies. Biochem Cell Biol.

[11] Mijanovic O, Brankovic A, Panin AN, Savchuk S, Timashev P, Ulasov I (2019). Cathepsin B: A sellsword of cancer progression. Cancer Lett.

[12] Pislar A, Jewett A, Kos J (2018). Cysteine cathepsins: Their biological and molecular significance in cancer stem cells. Semin Cancer Biol.

[13] Li J, Maeji M, Balboula AZ, Aboelenain M, Fujii T, Moriyasu S (2020). Dynamic status of lysosomal cathepsin in bovine oocytes and preimplantation embryos. J Reprod Dev.

[14] Mitrović A, Mirković B, Sosič I, Gobec S, Kos J (2016). Inhibition of endopeptidase and exopeptidase activity of cathepsin B impairs extracellular matrix degradation and tumour invasion. Biol Chem.

[15] Liu CL, Guo J, Zhang X, Sukhova GK, Libby P, Shi GP (2018). Cysteine protease cathepsins in cardiovascular disease: from basic research to clinical trials. Nat Rev Cardiol.

[16] Lawrence RE, Zoncu R (2019). The lysosome as a cellular centre for signalling, metabolism and quality control. Nat Cell Biol.

[17] Lambeth TR, Dai Z, Zhang Y, Julian RR (2021). A two-trick pony: lysosomal protease cathepsin B possesses surprising ligase activity. RSC Chem Biol.

[18] Hu YB, Dammer EB, Ren RJ, Wang G (2015). The endosomal-lysosomal system: from acidification and cargo sorting to neurodegeneration. Transl Neurodegener.

[19] Bohley P, Seglen PO (1992). Proteases and proteolysis in the lysosome. Experientia.

[20] Ha SD, Martins A, Khazaie K, Han J, Chan BM, Kim SO (2008). Cathepsin B is involved in the trafficking of TNF-alpha-containing vesicles to the plasma membrane in macrophages. J Immunol.

[21] Wang Y, Xi W, Zhang X, Bi X, Liu B, Zheng X (2022). CTSB promotes sepsis-induced acute kidney injury through activating mitochondrial apoptosis pathway. Front Immunol.

[22] Hamalisto S, Stahl JL, Favaro E, Yang Q, Liu B, Christoffersen L (2020). Spatially and temporally defined lysosomal leakage facilitates mitotic chromosome segregation. Nat Commun.

[23] Gocheva V, Zeng W, Ke D, Klimstra D, Reinheckel T, Peters C (2006). Distinct roles for cysteine cathepsin genes in multistage tumorigenesis. Genes Dev.

[24] Hanahan D, Coussens LM (2012). Accessories to the crime: functions of cells recruited to the tumor microenvironment. Cancer Cell.

[25] Skrzydlewska E, Sulkowska M, Koda M, Sulkowski S (2005). Proteolytic-antiproteolytic balance and its regulation in carcinogenesis. World J Gastroenterol.

[26] Masciocchi D, Gelain A, Villa S, Meneghetti F, Barlocco D (2011). a promising target for anticancer therapy. Future Med Chem.

[27] Chen C, Ahmad MJ, Ye T, Du C, Zhang X, Liang A (2021). Cathepsin B Regulates Mice Granulosa Cells' Apoptosis and Proliferation In Vitro. Int J Mol Sci.

[28] Li JX, Ding XM, Han S, Wang K, Jiao CY, Li XC (2018). mir-637 inhibits the proliferation of cholangiocarcinoma cell QBC939 through interfering CTSB expression. Eur Rev Med Pharmacol Sci.

[29] Bian B, Mongrain S, Cagnol S, Langlois M-J, Boulanger J, Bernatchez G (2016). Cathepsin B promotes colorectal tumorigenesis, cell invasion, and metastasis. Molecular Carcinogenesis.

[30] Gopinath S, Alapati K, Malla RR, Gondi CS, Mohanam S, Dinh DH (2011). Mechanism of p27 upregulation induced by downregulation of cathepsin B and uPAR in glioma. Mol Oncol.

[31] Česen MH, Pegan K, Spes A, Turk B (2012). Lysosomal pathways to cell death and their therapeutic applications. Exp Cell Res.

[32] Terman A, Gustafsson B, Brunk UT (2006). The lysosomal-mitochondrial axis theory of postmitotic aging and cell death. Chem Biol Interact.

[33] Guicciardi ME, Bronk SF, Werneburg NW, Yin XM, Gores GJ (2005). Bid is upstream of lysosome-mediated caspase 2 activation in tumor necrosis factor alpha-induced hepatocyte apoptosis. Gastroenterology.

[34] Chwieralski CE, Welte T, Buhling F (2006). Cathepsin-regulated apoptosis. Apoptosis.

[35] Tardy C, Codogno P, Autefage H, Levade T, Andrieu-Abadie N (2006). Lysosomes and lysosomal proteins in cancer cell death (new players of an old struggle). Biochim Biophys Acta.

[36] Werneburg N, Guicciardi ME, Yin XM, Gores GJ (2004). TNF-alpha-mediated lysosomal permeabilization is FAN and caspase 8/Bid dependent. Am J Physiol Gastrointest Liver Physiol.

[37] Guicciardi ME, Deussing J, Miyoshi H, Bronk SF, Svingen PA, Peters C (2000). Cathepsin B contributes to TNF-alpha-mediated hepatocyte apoptosis by promoting mitochondrial release of cytochrome c. J Clin Invest.

[38] Guicciardi ME, Miyoshi H, Bronk SF, Gores GJ (2001). Cathepsin B knockout mice are resistant to tumor necrosis factor-alpha-mediated hepatocyte apoptosis and liver injury: implications for therapeutic applications. Am J Pathol.

[39] Hishita T, Tada-Oikawa S, Tohyama K, Miura Y, Nishihara T, Tohyama Y (2001). Caspase-3 activation by lysosomal enzymes in cytochrome c-independent apoptosis in myelodysplastic syndrome-derived cell line P39. Cancer Res.

[40] Bröker LE, Huisman C, Span SW, Rodriguez JA, Kruyt FA, Giaccone G (2004). Cathepsin B mediates caspase-independent cell death induced by microtubule stabilizing agents in non-small cell lung cancer cells. Cancer Res.

[41] Foghsgaard L, Wissing D, Mauch D, Lademann U, Bastholm L, Boes M (2001). Cathepsin B acts as a dominant execution protease in tumor cell apoptosis induced by tumor necrosis factor. J Cell Biol.

[42] Malla RR, Gopinath S, Alapati K, Gorantla B, Gondi CS, Rao JS (2012). uPAR and cathepsin B inhibition enhanced radiation-induced apoptosis in gliomainitiating cells. Neuro Oncol.

[43] Talukdar R, Sareen A, Zhu H, Yuan Z, Dixit A, Cheema H (2016). Release of Cathepsin B in Cytosol Causes Cell Death in Acute Pancreatitis. Gastroenterology.

[44] Nagaraj NS, Vigneswaran N, Zacharias W (2006). Cathepsin B mediates TRAIL-induced apoptosis in oral cancer cells. J Cancer Res Clin Oncol.

[45] Gondi CS, Kandhukuri N, Kondraganti S, Gujrati M, Olivero WC, Dinh DH (2006). RNA interference-mediated simultaneous down-regulation of urokinase-type plasminogen activator receptor and cathepsin B induces caspase-8-mediated apoptosis in SNB19 human glioma cells. Mol Cancer Ther.

[46] Byrne SM, Aucher A, Alyahya S, Elder M, Olson ST, Davis DM (2012). Cathepsin B controls the persistence of memory CD8+ T lymphocytes. J Immunol.

[47] Kreuzaler PA, Staniszewska AD, Li W, Omidvar N, Kedjouar B, Turkson J (2011). Stat3 controls lysosomal-mediated cell death in vivo. Nat Cell Biol.

[48] Yadati T, Houben T, Bitorina A, Shiri-Sverdlov R (2020). The Ins and Outs of Cathepsins: Physiological Function and Role in Disease Management. Cells.

[49] Iwama H, Mehanna S, Imasaka M, Hashidume S, Nishiura H, Yamamura KI (2021). Cathepsin B and D deficiency in the mouse pancreas induces impaired autophagy and chronic pancreatitis. Sci Rep.

[50] Huai J, Vögtle FN, Jöckel L, Li Y, Kiefer T, Ricci JE (2013). TNFα-induced lysosomal membrane permeability is downstream of MOMP and triggered by caspase-mediated NDUFS1 cleavage and ROS formation. J Cell Sci.

[51] Xu J, Jiang Y, Wang J, Shi X, Liu Q, Liu Z (2014). Macrophage endocytosis of high-mobility group box 1 triggers pyroptosis. Cell Death Differ.

[52] Chen N, Ou Z, Zhang W, Zhu X, Li P, Gong J (2018). Cathepsin B regulates non-canonical NLRP3 inflammasome pathway by modulating activation of caspase-11 in Kupffer cells. Cell Prolif.

[53] Dixon SJ, Lemberg KM, Lamprecht MR, Skouta R, Zaitsev EM, Gleason CE (2012). Ferroptosis: an iron-dependent form of nonapoptotic cell death. Cell.

[54] Kuang F, Liu J, Li C, Kang R, Tang D (2020). Cathepsin B is a mediator of organelle-specific initiation of ferroptosis. Biochem Biophys Res Commun.

[55] Gao H, Bai Y, Jia Y, Zhao Y, Kang R, Tang D (2018). Ferroptosis is a lysosomal cell death process. Biochem Biophys Res Commun.

[56] Lee DH, Park SH, Ahn J, Hong SP, Lee E, Jang YJ (2021). Mir214-3p and Hnf4a/Hnf4α reciprocally regulate Ulk1 expression and autophagy in nonalcoholic hepatic steatosis. Autophagy.

[57] Qi X, Man SM, Malireddi RK, Karki R, Lupfer C, Gurung P (2016). Cathepsin B modulates lysosomal biogenesis and host defense against Francisella novicida infection. J Exp Med.

[58] Jiang L, Qiu T, Yao X, Chen H, Yao K, Sun X (2021). MEHP induces pyroptosis and autophagy alternation by cathepsin B activation in INS-1 cells. Environ Sci Pollut Res Int.

[59] Moon HY, Becke A, Berron D, Becker B, Sah N, Benoni G (2016). Running-Induced Systemic Cathepsin B Secretion Is Associated with Memory Function. Cell Metab.

[60] Mueller-Steiner S, Zhou Y, Arai H, Roberson ED, Sun B, Chen J (2006). Antiamyloidogenic and neuroprotective functions of cathepsin B: implications for Alzheimer's disease. Neuron.

[61] Hook G, Yu J, Toneff T, Kindy M, Hook V (2014). Brain pyroglutamate amyloid-β is produced by cathepsin B and is reduced by the cysteine protease inhibitor E64d, representing a potential Alzheimer's disease therapeutic. J Alzheimers Dis.

[62] Hook V, Toneff T, Bogyo M, Greenbaum D, Medzihradszky KF, Neveu J (2005). Inhibition of cathepsin B reduces beta-amyloid production in regulated secretory vesicles of neuronal chromaffin cells: evidence for cathepsin B as a candidate beta-secretase of Alzheimer's disease. Biol Chem.

[63] Ge J, Zhao G, Chen R, Li S, Wang S, Zhang X (2006). Enhanced myocardial cathepsin B expression in patients with dilated cardiomyopathy. Eur J Heart Fail.

[64] Cheng XW, Shi GP, Kuzuya M, Sasaki T, Okumura K, Murohara T (2012). Role for cysteine protease cathepsins in heart disease: focus on biology and mechanisms with clinical implication. Circulation.

[65] Müller AL, Dhalla NS (2012). Role of various proteases in cardiac remodeling and progression of heart failure. Heart Fail Rev.

[66] Osonoi Y, Mita T, Azuma K, Nakajima K, Masuyama A, Goto H (2018). Defective autophagy in vascular smooth muscle cells enhances cell death and atherosclerosis. Autophagy.

[67] Dinnes DL, White MY, Kockx M, Traini M, Hsieh V, Kim MJ (2016). Human macrophage cathepsin B-mediated C-terminal cleavage of apolipoprotein A-I at Ser228 severely impairs antiatherogenic capacity. Faseb j.

[68] Shalia KK, Mashru MR, Shah VK, Soneji SL, Payannavar S (2012). Levels of cathepsins in acute myocardial infarction. Indian Heart J.

[69] Larionov A, Dahlke E, Kunke M, Zanon Rodriguez L, Schiessl IM, Magnin JL (2019). Cathepsin B increases ENaC activity leading to hypertension early in nephrotic syndrome. J Cell Mol Med.

[70] Chai T, Tian M, Yang X, Qiu Z, Lin X, Chen L (2022). Association of Circulating Cathepsin B Levels With Blood Pressure and Aortic Dilation. Front Cardiovasc Med.

[71] Wang Y, Jia L, Shen J, Wang Y, Fu Z, Su SA (2018). Cathepsin B aggravates coxsackievirus B3-induced myocarditis through activating the inflammasome and promoting pyroptosis. PLoS Pathog.

[72] Wu QQ, Xu M, Yuan Y, Li FF, Yang Z, Liu Y (2015). Cathepsin B deficiency attenuates cardiac remodeling in response to pressure overload via TNF-α/ASK1/JNK pathway. Am J Physiol Heart Circ Physiol.

[73] Gornicka A, Fettig J, Eguchi A, Berk MP, Thapaliya S, Dixon LJ (2012). Adipocyte hypertrophy is associated with lysosomal permeability both in vivo and in vitro: role in adipose tissue inflammation. Am J Physiol Endocrinol Metab.

[74] Mizunoe Y, Sudo Y, Okita N, Hiraoka H, Mikami K, Narahara T (2017). Involvement of lysosomal dysfunction in autophagosome accumulation and early pathologies in adipose tissue of obese mice. Autophagy.

[75] Guo H, Zhao M, Qiu X, Deis JA, Huang H, Tang QQ (2016). Niemann-Pick type C2 deficiency impairs autophagy-lysosomal activity, mitochondrial function, and TLR signaling in adipocytes. J Lipid Res.

[76] Zhang ZY, Mai Y, Yang H, Dong PY, Zheng XL, Yang GS (2014). CTSB promotes porcine preadipocytes differentiation by degrading fibronectin and attenuating the Wnt/β-catenin signaling pathway. Mol Cell Biochem.

[77] Zheng T, Zhu Z, Wang Z, Homer RJ, Ma B, Riese RJ Jr (2000). Inducible targeting of IL-13 to the adult lung causes matrix metalloproteinase- and cathepsin-dependent emphysema. J Clin Invest.

[78] Burnett D, Abrahamson M, Devalia JL, Sapsford RJ, Davies RJ, Buttle DJ (1995). Synthesis and secretion of procathepsin B and cystatin C by human bronchial epithelial cells in vitro: modulation of cathepsin B activity by neutrophil elastase. Arch Biochem Biophys.

[79] Geraghty P, Rogan MP, Greene CM, Boxio RM, Poiriert T, O'Mahony M (2007). Neutrophil elastase up-regulates cathepsin B and matrix metalloprotease-2 expression. J Immunol.

[80] Walsh DE, Greene CM, Carroll TP, Taggart CC, Gallagher PM, O'Neill SJ (2001). Interleukin-8 up-regulation by neutrophil elastase is mediated by MyD88/IRAK/TRAF-6 in human bronchial epithelium. J Biol Chem.

[81] Mohamed MM, Sloane BF (2006). Cysteine cathepsins: multifunctional enzymes in cancer. Nat Rev Cancer.

[82] Podgorski I, Sloane BF (2003). Cathepsin B and its role(s) in cancer progression. Biochem Soc Symp.

[83] Carmeliet P (2003). Angiogenesis in health and disease. Nat Med.

[84] Im E, Venkatakrishnan A, Kazlauskas A (2005). Cathepsin B regulates the intrinsic angiogenic threshold of endothelial cells. Mol Biol Cell.

[85] Joyce JA, Baruch A, Chehade K, Meyer-Morse N, Giraudo E, Tsai FY (2004). Cathepsin cysteine proteases are effectors of invasive growth and angiogenesis during multistage tumorigenesis. Cancer Cell.

[86] Malla RR, Gopinath S, Gondi CS, Alapati K, Dinh DH, Gujrati M (2011). Cathepsin B and uPAR knockdown inhibits tumor-induced angiogenesis by modulating VEGF expression in glioma. Cancer Gene Ther.

[87] Yanamandra N, Gumidyala KV, Waldron KG, Gujrati M, Olivero WC, Dinh DH (2004). Blockade of cathepsin B expression in human glioblastoma cells is associated with suppression of angiogenesis. Oncogene.

[88] Sevenich L, Werner F, Gajda M, Schurigt U, Sieber C, Müller S (2011). Transgenic expression of human cathepsin B promotes progression and metastasis of polyoma-middle-T-induced breast cancer in mice. Oncogene.

[89] Kast RE (2010). Glioblastoma invasion, cathepsin B, and the potential for both to be inhibited by auranofin, an old anti-rheumatoid arthritis drug. Cent Eur Neurosurg.

[90] Kong X, Ma W, Li Y, Wang Y, Guan J, Gao J (2015). Does Tenascin have Clinical Implications in Pathological Grade of Glioma Patients?: A Systematic Meta-Analysis. Medicine (Baltimore).

[91] Kostoulas G, Lang A, Nagase H, Baici A (1999). Stimulation of angiogenesis through cathepsin B inactivation of the tissue inhibitors of matrix metalloproteinases. FEBS Lett.

[92] Koblinski JE, Ahram M, Sloane BF (2000). Unraveling the role of proteases in cancer. Clin Chim Acta.

[93] Singh R, Mishra MK, Aggarwal H (2017). Inflammation, Immunity, and Cancer. Mediators Inflamm.

[94] Baghban R, Roshangar L, Jahanban-Esfahlan R, Seidi K, Ebrahimi-Kalan A, Jaymand M (2020). Tumor microenvironment complexity and therapeutic implications at a glance. Cell Commun Signal.

[95] Gocheva V, Wang HW, Gadea BB, Shree T, Hunter KE, Garfall AL (2010). IL-4 induces cathepsin protease activity in tumor-associated macrophages to promote cancer growth and invasion. Genes Dev.

[96] Yoon MC, Solania A, Jiang Z, Christy MP, Podvin S, Mosier C (2021). Selective Neutral pH Inhibitor of Cathepsin B Designed Based on Cleavage Preferences at Cytosolic and Lysosomal pH Conditions. ACS Chem Biol.

[97] Giusti I, D'Ascenzo S, Millimaggi D, Taraboletti G, Carta G, Franceschini N (2008). Cathepsin B mediates the pH-dependent proinvasive activity of tumor-shed microvesicles. Neoplasia.

[98] Mason SD, Joyce JA (2011). Proteolytic networks in cancer. Trends Cell Biol.

[99] Chen BJ, Tang YJ, Tang YL, Liang XH (2019). What makes cells move: Requirements and obstacles for leader cells in collective invasion. Exp Cell Res.

[100] Vasiljeva O, Papazoglou A, Krüger A, Brodoefel H, Korovin M, Deussing J (2006). Tumor cell-derived and macrophage-derived cathepsin B promotes progression and lung metastasis of mammary cancer. Cancer Res.

[101] Sylven B, Malmgren H (1957). The histological distribution of proteinase and peptidase activity in solid tumor transplants; a histochemical study on the enzymic characteristics of the different tumor cell types. Acta Radiol Suppl.

[102] Sloane BF, Yan S, Podgorski I, Linebaugh BE, Cher ML, Mai J (2005). Cathepsin B and tumor proteolysis: contribution of the tumor microenvironment. Semin Cancer Biol.

[103] Buck MR, Karustis DG, Day NA, Honn KV, Sloane BF (1992). Degradation of extracellular-matrix proteins by human cathepsin B from normal and tumour tissues. Biochem J.

[104] Su SC, Lin CW, Yang WE, Fan WL, Yang SF (2016). The urokinase-type plasminogen activator (uPA) system as a biomarker and therapeutic target in human malignancies. Expert Opin Ther Targets.

[105] Ruan J, Zheng H, Rong X, Rong X, Zhang J, Fang W (2016). Over-expression of cathepsin B in hepatocellular carcinomas predicts poor prognosis of HCC patients. Mol Cancer.

[106] Xu ZZ, Xiu P, Lv JW, Wang FH, Dong XF, Liu F (2015). Integrin αvβ3 is required for cathepsin B-induced hepatocellular carcinoma progression. Mol Med Rep.

[107] Peng S, Yang Q, Li H, Pan Y, Wang J, Hu P (2021). CTSB Knockdown Inhibits Proliferation and Tumorigenesis in HL-60 Cells. Int J Med Sci.

[108] Victor BC, Anbalagan A, Mohamed MM, Sloane BF, Cavallo-Medved D (2011). Inhibition of cathepsin B activity attenuates extracellular matrix degradation and inflammatory breast cancer invasion. Breast Cancer Res.

[109] Nouh MA, Mohamed MM, El-Shinawi M, Shaalan MA, Cavallo-Medved D, Khaled HM (2011). Cathepsin B: a potential prognostic marker for inflammatory breast cancer. J Transl Med.

[110] Mohamed MM, Cavallo-Medved D, Sloane BF (2008). Human monocytes augment invasiveness and proteolytic activity of inflammatory breast cancer. Biol Chem.

[111] Yamaguchi H, Takeo Y, Yoshida S, Kouchi Z, Nakamura Y, Fukami K (2009). Lipid rafts and caveolin-1 are required for invadopodia formation and extracellular matrix degradation by human breast cancer cells. Cancer Res.

[112] Cavallo-Medved D, Mai J, Dosescu J, Sameni M, Sloane BF (2005). Caveolin-1 mediates the expression and localization of cathepsin B, pro-urokinase plasminogen activator and their cell-surface receptors in human colorectal carcinoma cells. J Cell Sci.

[113] Cavallo-Medved D, Dosescu J, Linebaugh BE, Sameni M, Rudy D, Sloane BF (2003). Mutant K-ras regulates cathepsin B localization on the surface of human colorectal carcinoma cells. Neoplasia.

[114] Wu JB, Yin L, Shi C, Li Q, Duan P, Huang JM (2017). MAOA-Dependent Activation of Shh-IL6-RANKL Signaling Network Promotes Prostate Cancer Metastasis by Engaging Tumor-Stromal Cell Interactions. Cancer Cell.

[115] Mai J, Sameni M, Mikkelsen T, Sloane BF (2002). Degradation of extracellular matrix protein tenascin-C by cathepsin B: an interaction involved in the progression of gliomas. Biol Chem.

[116] Shimizu A, Nakayama H, Wang P, König C, Akino T, Sandlund J (2013). Netrin-1 promotes glioblastoma cell invasiveness and angiogenesis by multiple pathways including activation of RhoA, cathepsin B, and cAMP-response element-binding protein. J Biol Chem.

[117] Mai J, Finley RL Jr, Waisman DM, Sloane BF (2000). Human procathepsin B interacts with the annexin II tetramer on the surface of tumor cells. J Biol Chem.

[118] Mai J, Waisman DM, Sloane BF (2000). Cell surface complex of cathepsin B/annexin II tetramer in malignant progression. Biochim Biophys Acta.

[119] Wang YX, Lv H, Li ZX, Li C, Wu XY (2012). Effect of shRNA mediated down-regulation of Annexin A2 on biological behavior of human lung adencarcinoma cells A549. Pathol Oncol Res.

[120] Vasiljeva O, Korovin M, Gajda M, Brodoefel H, Bojic L, Krüger A (2008). Reduced tumour cell proliferation and delayed development of high-grade mammary carcinomas in cathepsin B-deficient mice. Oncogene.

[121] Liu G, Feng S, Jia L, Wang C, Fu Y, Luo Y (2018). Lung fibroblasts promote metastatic colonization through upregulation of stearoyl-CoA desaturase 1 in tumor cells. Oncogene.

[122] Chen JY, Chen WN, Liu LL, Lin WS, Jiao BY, Wu YL (2010). Hepatitis B spliced protein (HBSP) generated by a spliced hepatitis B virus RNA participates in abnormality of fibrin formation and functions by binding to fibrinogen γ chain. J Med Virol.

[123] Chen WN, Chen JY, Jiao BY, Lin WS, Wu YL, Liu LL (2012). Interaction of the hepatitis B spliced protein with cathepsin B promotes hepatoma cell migration and invasion. J Virol.

[124] Kumar A, Dhar S, Campanelli G, Butt NA, Schallheim JM, Gomez CR (2018). MTA1 drives malignant progression and bone metastasis in prostate cancer. Mol Oncol.

[125] Alapati K, Gopinath S, Malla RR, Dasari VR, Rao JS (2012). uPAR and cathepsin B knockdown inhibits radiation-induced PKC integrated integrin signaling to the cytoskeleton of glioma-initiating cells. Int J Oncol.

[126] Serrels A, Canel M, Brunton VG, Frame MC (2011). Src/FAK-mediated regulation of E-cadherin as a mechanism for controlling collective cell movement: insights from in vivo imaging. Cell Adh Migr.

[127] Wu JS, Li ZF, Wang HF, Yu XH, Pang X, Wu JB (2019). Cathepsin B defines leader cells during the collective invasion of salivary adenoid cystic carcinoma. Int J Oncol.

[128] Bengsch F, Buck A, Gunther SC, Seiz JR, Tacke M, Pfeifer D (2014). Cell type-dependent pathogenic functions of overexpressed human cathepsin B in murine breast cancer progression. Oncogene.

[129] Peng D, Fu M, Wang M, Wei Y, Wei X (2022). Targeting TGF-beta signal transduction for fibrosis and cancer therapy. Mol Cancer.

[130] Yin M, Soikkeli J, Jahkola T, Virolainen S, Saksela O, Hölttä E (2012). TGF-β signaling, activated stromal fibroblasts, and cysteine cathepsins B and L drive the invasive growth of human melanoma cells. Am J Pathol.

[131] Gogineni VR, Gupta R, Nalla AK, Velpula KK, Rao JS (2012). uPAR and cathepsin B shRNA impedes TGF-β1-driven proliferation and invasion of meningioma cells in a XIAP-dependent pathway. Cell Death Dis.

[132] Reisenauer A, Eickelberg O, Wille A, Heimburg A, Reinhold A, Sloane BF (2007). Increased carcinogenic potential of myeloid tumor cells induced by aberrant TGF-beta1-signaling and upregulation of cathepsin B. Biol Chem.

[133] Moustakas A, Heldin CH (2016). Mechanisms of TGFβ-Induced Epithelial-Mesenchymal Transition. J Clin Med.

[134] Yan S, Jane DT, Dufresne MJ, Sloane BF (2003). Transcription of cathepsin B in glioma cells: regulation by an E-box adjacent to the transcription initiation site. Biol Chem.

[135] Wellner U, Schubert J, Burk UC, Schmalhofer O, Zhu F, Sonntag A (2009). The EMT-activator ZEB1 promotes tumorigenicity by repressing stemness-inhibiting microRNAs. Nat Cell Biol.

[136] Mitrović A, Pečar Fonović U, Kos J (2017). Cysteine cathepsins B and X promote epithelial-mesenchymal transition of tumor cells. Eur J Cell Biol.

[137] Kasabova M, Joulin-Giet A, Lecaille F, Gilmore BF, Marchand-Adam S, Saidi A (2014). Regulation of TGF-β1-driven differentiation of human lung fibroblasts: emerging roles of cathepsin B and cystatin C. J Biol Chem.

[138] Kim EK, Song MJ, Jang HH, Chung YS (2020). Clinicopathologic Analysis of Cathepsin B as a Prognostic Marker of Thyroid Cancer. Int J Mol Sci.

[139] Jiang Y, Woosley AN, Sivalingam N, Natarajan S, Howe PH (2016). Cathepsin-B-mediated cleavage of Disabled-2 regulates TGF-β-induced autophagy. Nat Cell Biol.

[140] Kryczka J, Papiewska-Pajak I, Kowalska MA, Boncela J (2019). Cathepsin B Is Upregulated and Mediates ECM Degradation in Colon Adenocarcinoma HT29 Cells Overexpressing Snail. Cells.

[141] Gopinath S, Malla R, Alapati K, Gorantla B, Gujrati M, Dinh DH (2013). Cathepsin B and uPAR regulate self-renewal of glioma-initiating cells through GLI-regulated Sox2 and Bmi1 expression. Carcinogenesis.

[142] Featherston T, Marsh RW, van Schaijik B, Brasch HD, Tan ST, Itinteang T (2017). Expression and Localization of Cathepsins B, D, and G in Two Cancer Stem Cell Subpopulations in Moderately Differentiated Oral Tongue Squamous Cell Carcinoma. Front Med (Lausanne).

[143] Tang KD, Liu J, Jovanovic L, An J, Hill MM, Vela I (2016). Adipocytes promote prostate cancer stem cell self-renewal through amplification of the cholecystokinin autocrine loop. Oncotarget.

[144] Featherston T, Brasch HD, Siljee SD, van Schaijik B, Patel J, de Jongh J (2020). Cancer Stem Cells in Head and Neck Cutaneous Squamous Cell Carcinoma Express Cathepsins. Plast Reconstr Surg Glob Open.

[145] Fujimoto T, Tsunedomi R, Matsukuma S, Yoshimura K, Oga A, Fujiwara N (2021). Cathepsin B is highly expressed in pancreatic cancer stem-like cells and is associated with patients' surgical outcomes. Oncol Lett.

[146] Zhang S, Mercado-Uribe I, Liu J (2013). Generation of erythroid cells from fibroblasts and cancer cells in vitro and in vivo. Cancer Lett.

[147] Zhang D, Wang Y, Zhang S (2014). Asymmetric cell division in polyploid giant cancer cells and low eukaryotic cells. Biomed Res Int.

[148] Zhang D, Yang X, Yang Z, Fei F, Li S, Qu J (2017). Daughter Cells and Erythroid Cells Budding from PGCCs and Their Clinicopathological Significances in Colorectal Cancer. J Cancer.

[149] Toupin NP, Arora K, Shrestha P, Peterson JA, Fischer LJ, Rajagurubandara E (2019). BODIPY-Caged Photoactivated Inhibitors of Cathepsin B Flip the Light Switch on Cancer Cell Apoptosis. ACS Chem Biol.

[150] Osmak M, Svetić B, Gabrijelcić-Geiger D, Skrk J (2001). Drug-resistant human laryngeal carcinoma cells have increased levels of cathepsin B. Anticancer Res.

[151] Shim MK, Moon Y, Yang S, Kim J, Cho H, Lim S (2020). Cancer-specific drug-drug nanoparticles of pro-apoptotic and cathepsin B-cleavable peptide-conjugated doxorubicin for drug-resistant cancer therapy. Biomaterials.

[152] Bechara A, Barbosa CM, Paredes-Gamero EJ, Garcia DM, Silva LS, Matsuo AL (2014). Palladacycle (BPC) antitumour activity against resistant and metastatic cell lines: the relationship with cytosolic calcium mobilisation and cathepsin B activity. Eur J Med Chem.

[153] Jiang H, Acharya C, An G, Zhong M, Feng X, Wang L (2016). SAR650984 directly induces multiple myeloma cell death via lysosomal-associated and apoptotic pathways, which is further enhanced by pomalidomide. Leukemia.

[154] Puissant A, Colosetti P, Robert G, Cassuto JP, Raynaud S, Auberger P (2010). Cathepsin B release after imatinib-mediated lysosomal membrane permeabilization triggers BCR-ABL cleavage and elimination of chronic myelogenous leukemia cells. Leukemia.

[155] Zou ZZ, Nie PP, Li YW, Hou BX, Rui L, Shi XP (2017). Synergistic induction of apoptosis by salinomycin and gefitinib through lysosomal and mitochondrial dependent pathway overcomes gefitinib resistance in colorectal cancer. Oncotarget.

[156] Ho KH, Cheng CH, Chou CM, Chen PH, Liu AJ, Lin CW (2019). miR-140 targeting CTSB signaling suppresses the mesenchymal transition and enhances temozolomide cytotoxicity in glioblastoma multiforme. Pharmacol Res.

[157] Dyshlovoy SA, Hauschild J, Amann K, Tabakmakher KM, Venz S, Walther R (2015). Marine alkaloid Monanchocidin a overcomes drug resistance by induction of autophagy and lysosomal membrane permeabilization. Oncotarget.

[158] Chowdhury KD, Sarkar A, Chatterjee S, Patra D, Sengupta D, Banerjee S (2019). Cathepsin B mediated scramblase activation triggers cytotoxicity and cell cycle arrest by andrographolide to overcome cellular resistance in cisplatin resistant human hepatocellular carcinoma HepG2 cells. Environ Toxicol Pharmacol.

[159] Jubeh B, Breijyeh Z, Karaman R (2020). Antibacterial Prodrugs to Overcome Bacterial Resistance. Molecules.

[160] Cho H, Shim MK, Yang S, Song S, Moon Y, Kim J (2021). Cathepsin B-Overexpressed Tumor Cell Activatable Albumin-Binding Doxorubicin Prodrug for Cancer-Targeted Therapy. Pharmaceutics.

[161] Herceg V, Bouilloux J, Janikowska K, Allémann E, Lange N (2020). Cathepsin B-Cleavable Cyclopeptidic Chemotherapeutic Prodrugs. Molecules.

[162] Kim J, Shim MK, Cho YJ, Jeon S, Moon Y, Choi J (2021). The safe and effective intraperitoneal chemotherapy with cathepsin B-specific doxorubicin prodrug nanoparticles in ovarian cancer with peritoneal carcinomatosis. Biomaterials.

[163] Li YY, Fang J, Ao GZ (2017). Cathepsin B and L inhibitors: a patent review (2010 - present). Expert Opin Ther Pat.

[164] Dikovskaya MA, Trunov AN, Chernykh VV, Korolenko TA (2013). Cystatin C and lactoferrin concentrations in biological fluids as possible prognostic factors in eye tumor development. Int J Circumpolar Health.

[165] Withana NP, Blum G, Sameni M, Slaney C, Anbalagan A, Olive MB (2012). Cathepsin B inhibition limits bone metastasis in breast cancer. Cancer Res.

[166] Ansari MA, Nadeem A, Alshammari MA, Attia SM, Bakheet SA, Khan MR (2022). Cathepsin B inhibitor alleviates Th1, Th17, and Th22 transcription factor signaling dysregulation in experimental autoimmune encephalomyelitis. Exp Neurol.

[167] Pandey G, Bakhshi S, Kumar M, Thakur B, Jain P, Kaur P (2019). Prognostic and therapeutic relevance of cathepsin B in pediatric acute myeloid leukemia. Am J Cancer Res.

[168] Hook GR, Yu J, Sipes N, Pierschbacher MD, Hook V, Kindy MS (2014). The cysteine protease cathepsin B is a key drug target and cysteine protease inhibitors are potential therapeutics for traumatic brain injury. J Neurotrauma.

[169] Buttle DJ, Murata M, Knight CG, Barrett AJ (1992). CA074 methyl ester: a proinhibitor for intracellular cathepsin B. Arch Biochem Biophys.

[170] Wang J, Wang L, Zhang X, Xu Y, Chen L, Zhang W (2021). Cathepsin B aggravates acute pancreatitis by activating the NLRP3 inflammasome and promoting the caspase-1-induced pyroptosis. Int Immunopharmacol.

[171] Feng Y, Ni L, Wang Q (2013). Administration of cathepsin B inhibitor CA-074Me reduces inflammation and apoptosis in polymyositis. J Dermatol Sci.

[172] Yan BZ, Wang W, Chen LY, Bi MR, Lu YJ, Li BX (2009). Role of cathepsin B-mediated apoptosis in fulminant hepatic failure in mice. World J Gastroenterol.

[173] Rudzińska M, Parodi A, Soond SM, Vinarov AZ, Korolev DO, Morozov AO (2019). The Role of Cysteine Cathepsins in Cancer Progression and Drug Resistance. Int J Mol Sci.

[174] Rawlings ND, Barrett AJ, Thomas PD, Huang X, Bateman A, Finn RD (2018). The MEROPS database of proteolytic enzymes, their substrates and inhibitors in 2017 and a comparison with peptidases in the PANTHER database. Nucleic Acids Res.

[175] Sinha AA, Morgan JL, Wood N, Betre K, Reddy A, Wilson MJ (2007). Heterogeneity of cathepsin B and stefin A expression in Gleason pattern 3+3 (score 6) prostate cancer needle biopsies. Anticancer Res.

[176] Duivenvoorden HM, Rautela J, Edgington-Mitchell LE, Spurling A, Greening DW, Nowell CJ (2017). Myoepithelial cell-specific expression of stefin A as a suppressor of early breast cancer invasion. J Pathol.

[177] Li C, Chen L, Wang J, Zhang L, Tang P, Zhai S (2011). Expression and clinical significance of cathepsin B and stefin A in laryngeal cancer. Oncol Rep.

[178] Li W, Ding F, Zhang L, Liu Z, Wu Y, Luo A (2005). Overexpression of stefin A in human esophageal squamous cell carcinoma cells inhibits tumor cell growth, angiogenesis, invasion, and metastasis. Clin Cancer Res.

[179] Ma Y, Chen Y, Li Y, Grün K, Berndt A, Zhou Z (2018). Cystatin A suppresses tumor cell growth through inhibiting epithelial to mesenchymal transition in human lung cancer. Oncotarget.

[180] Wang N, Yuan Y, Bai X, Han W, Han L, Qing B (2020). Association of cathepsin B and cystatin C with an age-related pulmonary subclinical state in a healthy Chinese population. Ther Adv Respir Dis.

[181] Illy C, Quraishi O, Wang J, Purisima E, Vernet T, Mort JS (1997). Role of the occluding loop in cathepsin B activity. J Biol Chem.

[182] Zore I, Krasovec M, Cimerman N, Kuhelj R, Werle B, Nielsen HJ (2001). Cathepsin B/cystatin C complex levels in sera from patients with lung and colorectal cancer. Biol Chem.

[183] Monsouvanh A, Proungvitaya T, Limpaiboon T, Wongkham C, Wongkham S, Luvira V (2014). Serum cathepsin B to cystatin C ratio as a potential marker for the diagnosis of cholangiocarcinoma. Asian Pac J Cancer Prev.

[184] Yan Y, Zhou K, Wang L, Wang F, Chen X, Fan Q (2017). Clinical significance of serum cathepsin B and cystatin C levels and their ratio in the prognosis of patients with esophageal cancer. Onco Targets Ther.

[185] Han H, Li J, Feng X, Zhou H, Guo S, Zhou W (2017). Autophagy-related genes are induced by histone deacetylase inhibitor suberoylanilide hydroxamic acid via the activation of cathepsin B in human breast cancer cells. Oncotarget.

[186] Yano M, Hirai K, Naito Z, Yokoyama M, Ishiwata T, Shiraki Y (2001). Expression of cathepsin B and cystatin C in human breast cancer. Surg Today.

[187] Kim JT, Lee SJ, Kang MA, Park JE, Kim BY, Yoon DY (2013). Cystatin SN neutralizes the inhibitory effect of cystatin C on cathepsin B activity. Cell Death Dis.

[188] Oh SS, Park S, Lee KW, Madhi H, Park SG, Lee HG (2017). Extracellular cystatin SN and cathepsin B prevent cellular senescence by inhibiting abnormal glycogen accumulation. Cell Death Dis.

